# An Explanation of How the High Frequency Violet Ray Apparatus Produces Its Effects

**Published:** 1917-10

**Authors:** George H. Reed

**Affiliations:** Dental Surgeon, U. S. Navy


### AN EXPLANATION OF HOW THE HIGH FREQUENCY VIOLET RAY APPARATUS PRODUCES ITS EFFECTS

BY GEORGE H. REED, DENTAL SURGEON, U. S. NAVY

Within the past few years there has been placed on the market a violet ray high frequency electrical generator, designed for use in the treatment of various systemic dis orders in medicine, and especially adapted for use in dental offices for the reduction of local abscesses, diagnostic work and molecular massage.

While the manufacturers of this instrument claim much for it as a mechanical agent in the reduction of local swellings, its value in sterilization and its ozone producing properties, all of which claims seem to be substantiated by its use, there is little literature available as to the definite action of this high frequency current on the tissues in producing these effects, and an absence of concrete information as to just what the violet ray is, how it is produced and the molecular changes brought about by its employment.

### DUBOIS RAYMOND THEORY

The DuBois Raymond theory presupposes the existence of electrical ions in muscular tissue, and this theory is substantiated by a laborious series of experiments, all considered at length in physiology and establishing the fact that each muscle has within itself these ions or myomeres, each with its positive and negative pole. These experiments have been conducted with galvanic and faradic currents.

### THE VIOLET RAY

The violet ray is the ordinary illuminating current, in which the voltage has been "stepped up," and is produced in a vacuum by high tension at a very low amperage. While the force of the current is extremely large, its volume is extremely small. A force of 50,000 volts is a low voltage to use in ordinary treatments with the violet ray apparatus, and this force can be applied by the operator by reason of its high frequency, the tissues not being able to react to the strength of the charge before it is withdrawn and another substituted. A favorite illustration of this is that of a passenger riding on a fast train passing a picket fence. The eye is not able to retain the picture of each individual picket, the fence having the appearance of being a solid board or only a blur on the landscape, depending upon the distance between the pickets of the fence and the speed of the train.

It is the reverse of the discovery that the retina of the eye retains an impression of a picture after the picture has been removed and another substituted in its place which is responsible for the moving picture.

The average dentist, on his acquisition of a violet ray machine, can not find the ground connection with which the ordinary electrical apparatus is always equipped. The explanation is simple; there is none. The current goes back into the generator from which it comes, the apparatus having both the cathode and anode within itself. This explains why it is not necessary to have positive and negative hand electrodes for the patient to hold in order to direct the current through a certain path by means of the application of electrodes to each end of the path.

### APPLICATION OF THE VIOLET RAY

In applying the electrode to a spot on the face of the patient, the entire system receives the charge, the area of application being the driving point from which place the current enters the body and where it goes out again. The operator can place the glass electrode on his own person, and using his fingers in place of the electrode, treat the patient. The operator's body in this case acts as a conductor and receives the bulk of the charge, yet having enough remain to produce a spark of appreciable length from the ends of his fingers with which to reach the spot in the mouth of his patient inaccessible to the ordinary straight glass electrode. This method is sometimes adopted to avoid accidental contact of the electrode and lips of the patient which sometimes causes a shock and in all events reduces the strength of the charge.

The writer has found that where using the glass electrodes in contact with a wet surface, the effect is more marked and often too much heat is generated for comfort, especially with a cup-shaped electrode. The use of the operator's fingers on mucous surfaces in working inside the mouth of course rectifies this difficulty.

### STERILIZING EFFECT OF VIOLET RAY

The claim is advanced that the action of the violet ray will sterilize the part adjacent to the driving point. It seems to do this, and the best explanation of how this is accomplished is believed to be as follows: The tissues and blood contain a large amount of sodium chloride, the normal saline solution. When a charge of electricity is passed through a wet medium, electrolysis takes place and the sodium chloride is changed into sodium hyperchlorite, a powerful oxydizing agent and germicide, the action of which on tissues sterilizes the part.

In this chemical change there are several intermediate changes which take place and subsidiary products are found, but the final result is the production temporarily of sodium hyperchlorite, the sterilizing properties of which accomplish the result desired. It is not an instantaneous sterilization, and the time of application has much to do with its permanency. Its value in this respect has been demonstrated by the cure of chronic abscesses without root amputation.

The theory has also been advanced that the sodium salts of the tissues are decomposed into chlorine gas which is picked up on the anodes, and water which is picked up at the cathode, the water further undergoing chemical change and becoming $H_2O_2$, the chlorin gas acting as the sterilizing agent.

The value of the violet ray in reducing a swelling lies in the fact that the molecular bombardment of the tissues produced by the application of the ray starts up and diffuses the congested circulation which causes the swelling.

The high frequency generator produces an electric current that increases the body heat without increasing the temperature. It stimulates by the increased production of oxygen in the form of ozone and by the increased force and volume of the electrical content of muscle tissue itself. It loosens mechanically and chemically molecular energy, has a highly energizing effect locally and produces an increased tonicity of the part adjacent to the point of application.

In this connection, with the tissues in a passive state, not only are the energy producing properties stimulated, but the eliminative functions also, maintaining a normal balance. Diffusion of energy accompanied by the elimination of waste is attended also by a mildly soothing effect that seems to allay irritable nervous symptoms and produces a relaxation that inspires confidence on the part of the patient in the work of the operator. This nervousness is a no inconsiderable obstacle daily to be overcome in the average dental office.

### DIAGNOSTIC USE OF VIOLET RAY

The diagnostic value of the violet ray can be illustrated as follows: Many dentists waste much valuable time in trying to accurately determine whether or not the pulp in a questionable tooth is alive or dead. Application of a spark from an electrode will generally settle this matter in a moment's time, with only a slightly perceptible shock to the patient if the pulp be healthy. Dentists who have used the ray longer than the writer have informed him that this test is not infallible, but that in every case where the test has been made in this connection and no response felt, upon drilling into the tooth, if not completely dead, the pulp has been diseased and dying.

A summary of the value of the high frequency apparatus in the field of dentistry, as here outlined, appears to include qualities, stimulative, diagnostic, eliminative, sedative and germicidal, with certain caustic properties, as far as the writer can determine with no literature on the subject to aid him. Incidentally it is not the ray that accomplishes these things, but the charge of electricity. The ray would be equally valuable were it green or blue instead of violet.

The writer makes no claim that the so-called violet ray machine will accomplish all that is claimed for it, and correspondence with manufacturers of the instrument reveals the fact that some at least of the makers of the machine can not substantiate their advertising matter on the subject and in fact know little of what they are making. It is a fact, however, demonstrated by use at this office that it is a valuable help in the various ways enumerated.

There is plenty of literature published by the manufacturers of the violet ray instruments on how they are to be used and a list of dental ailments, with detailed instructions for their treatment as varied as are the machines made. This technic can best be developed by the use of the apparatus, and the choice of the machine may be based on the requirements of the operator. This article is intended as only a tentative explanation of the manner in which the results arrived at are produced.—*Dental Items of Interest* for June.

